# Association of Type D Personality and Anatomical Complexity as Predictors of Long-Term Mortality in Coronary Artery Disease: A Retrospective Case Study Based on Hospital Records

**DOI:** 10.3390/diseases14070244

**Published:** 2026-07-06

**Authors:** Omar Anwar Saleh Al Nakhebi, Răzvan Susan, Adriana Mihai, Gheorghe Adrian Bumbu, Florina Mădălina Mindru, Cristian Mornoș, Virgil-Radu Enătescu

**Affiliations:** 1 Doctoral School, “Victor Babeș” University of Medicine and Pharmacy, 300041 Timișoara, Romania; 2 Department of Family Medicine, Centre for Preventive Medicine, “Victor Babeș” University of Medicine and Pharmacy, Eftimie Murgu Square No. 2, 300041 Timișoara, Romania; 3 ME2 Discipline of Psychiatry, Psychiatry Clinic I, “George Emil Palade” University of Medicine, Pharmacy, Science and Technology, 540142 Târgu Mureș, Romania; 4 Department of Psychiatry, Faculty of Medicine and Pharmacy, University of Oradea, 410087 Oradea, Romania; abumbu@uoradea.ro (G.A.B.); mindru.florinamadalina@student.uoradea.ro (F.M.M.); 5 Institute of Cardiovascular Diseases, 300310 Timișoara, Romania; 6 Department of Cardiology 1, “Victor Babeș” University of Medicine and Pharmacy, 300041 Timișoara, Romania; 7 Discipline of Psychiatry, Department of Neurosciences, “Victor Babeș” University of Medicine and Pharmacy, 300041 Timișoara, Romania

**Keywords:** type D personality, SYNTAX score, anxiety paradox, SCL-90

## Abstract

**Background:** Traditional cardiovascular risk models often overlook “residual risk” driven by psychopathological factors. This study investigates the exploratory prognostic baseline associations of Type D personality (TDP) and specific symptomatic dimensions with long-term all-cause mortality in patients with coronary artery disease (CAD). **Methods:** We conducted a retrospective case study based on hospital records evaluating 221 patients with confirmed CAD. Anatomical complexity was quantified via the SYNTAX Score (SS). Psychological profiling utilized the DS14 scale for TDP and the SCL-90 for granular symptoms (depression, anxiety, and hostility). Mortality was analyzed over a mean follow-up of 1026 days using multivariate Cox proportional hazards models. **Results:** Over a mean follow-up of 1026 days, the overall all-cause mortality rate was 33.0% (n=73). TDP prevalence was 19.0% (n=42) and significantly correlated with higher anatomical complexity (SS: 26.21 vs. 15.49; p<0.001). In the adjusted psychological model, baseline anxiety symptom severity presented an exploratory, borderline relationship with survival (HR = 0.941; p=0.049), with the 95% confidence interval upper bound reaching the null threshold (1.000), suggesting a potential, hypothesis-generating “Anxiety Paradox”. The psychological model demonstrated variations in descriptive validation indices (C-index = 0.624) compared to a baseline model integrating trait metrics and anatomical severity (C-index = 0.527). Significant correlations were confirmed between SS and psychological distress (r=0.493). **Conclusions:** TDP components and granular psychological tracks show significant baseline associations with coronary anatomical distributions, while anxiety dimensions present an exploratory relationship with long-term survival. Given the lack of adjustment for major clinical determinants of mortality (such as age, comorbidities, or ventricular function), these findings must be interpreted strictly as hypothesis-generating and exploratory.

## 1. Introduction

Cardiovascular diseases (CVDs) remain the leading cause of global mortality, accounting for approximately 31.8% of all deaths worldwide [1,2]. Despite the optimization of clinical and pharmacological treatments focused on modifiable biological risk factors—such as hypertension, diabetes, and sedentary lifestyles—a significant burden of residual morbidity persists. This evidence suggests that traditional, purely biological medical models may be insufficient for a holistic understanding of patient prognosis [2,3,4,5]. Consequently, modern cardiology has progressively adopted a biopsychosocial perspective, recognizing that mental health and personality traits are not merely incidental comorbidities, but central and active components of cardiovascular pathophysiological processes [5,6].

Central to this exploration is the construct of Type D (distressed) personality (TDP), defined by the synergistic presence of Negative Affectivity (NA)—the propensity to experience emotional states of dysphoria and tension—and Social Inhibition (SI)—the tendency to suppress these emotions for fear of others’ judgment [7,8]. Unlike the Type A profile, characterized by external hyper-reactivity, Type D individuals internalize distress, often manifesting somatization phenomena. This phenotype has been associated with dysregulation of the hypothalamic–pituitary–adrenal (HPA) axis, sustained sympathetic activation, and elevated levels of pro-inflammatory mediators (CRP, IL-6, TNF-α), which promote endothelial dysfunction and increase the vulnerability of atherosclerotic plaques [4,9,10].

However, despite the established role of TDP in worsening cardiovascular outcomes, a critical gap remains in the literature regarding the distinction between stable “trait” vulnerability and acute, transient psychopathological symptomatology. While the anatomical severity of coronary artery disease (CAD) is routinely quantified via the SYNTAX Score, its ability to reflect true global risk is limited by the failure to integrate these “hidden drivers” of prognosis [4,11]. The scientific validity of a contemporary prognostic model lies, therefore, in its ability to demonstrate incremental predictive value: specifically, the capacity of psychological metrics to improve discriminant accuracy (C-index) beyond conventional clinical and anatomical parameters.

Furthermore, there is a need to investigate non-linear dynamics in the relationship between distress and survival. Contrary to the classical view that interprets all forms of psychic malaise as detrimental, the existence of an “Anxiety Paradox” is hypothesized. In this perspective, specific dimensions of the anxiety spectrum may translate into “proactive vigilance,” where a moderate level of concern favors greater treatment adherence and timely health-seeking behavior, potentially manifesting a paradoxically protective association with long-term survival. Based on these distinct gaps in current clinical knowledge, we formulated a multi-tiered hypothesis. We hypothesized that Type D personality and granular psychopathological tracks would demonstrate significant exploratory baseline associations with long-term mortality, and that their inclusion alongside anatomical complexity metrics could modify model discrimination. Concurrently, we hypothesized that distinct anxiety dimensions might present an exploratory, non-linear inverse relationship with mortality curves.

The primary objective of this study is to investigate these potential exploratory baseline associations of Type D personality and the symptomatic dimensions investigated via the SCL-90 (Symptom Checklist-90) with all-cause mortality. Through a retrospective analysis of a historical cohort of patients undergoing coronary evaluation and comprehensive psychometric profiling (DS14 and SCL-90), this research aims to determine whether the integration of these domains allows for the transcendence of the current unidirectional risk paradigm in favor of a cardiological management approach that is truly holistic, personalized, and based on biopsychosocial evidence.

## 2. Subjects and Methods

### 2.1. Ethical Approval and Regulatory Compliance

The research protocol, formally titled *“The Role of Psychological and Psychopathological Factors in Determining the Complexity of Coronary Artery Disease,”* underwent a rigorous review process and obtained approval from the Scientific Research Ethics Committee (CECS) at the “Victor Babeș” University of Medicine and Pharmacy, Timișoara (UMFVBT). The official authorization was issued under Aviz CECS No. 85/2025, subsequently revised on 12 March 2026. The study was conducted in full compliance with the ethical principles enshrined in the Declaration of Helsinki and the Good Clinical Practice (GCP) guidelines. To ensure maximum protection of privacy and the safeguarding of sensitive data, all information was treated in accordance with the General Data Protection Regulation (GDPR).

### 2.2. Study Design and Population

The research was configured as a retrospective case study based on institutional hospital records, initially based on a screening of 292 patients admitted to the Department of Cardiology. Through the application of rigorous eligibility criteria, a final subgroup of 221 patients with a confirmed diagnosis of CHD was selected for longitudinal analysis. Consequently, a total of 71 patients were excluded during the initial selection phase. Specifically, a total of 71 patients were excluded due to the following criteria: incomplete clinical or angiographic documentation (n = 34); missing baseline psychometric profiles (n = 21); and acute cognitive deficits, severe delirium, or minor consciousness impairments identified via bedside screening during admission (n = 16). The inclusion process required diagnostic confirmation of the pathology via coronary angiography and the completion of a baseline psychological assessment conducted at the time of diagnosis by the year 2015, including only subjects aged 18 years or older. Patients with incomplete clinical or angiographic documentation, as well as those lacking baseline psychometric data, were systematically excluded. Furthermore, patients suffering from acute cognitive deficits, severe delirium, or any milder forms of consciousness and cognitive impairments that could have compromised the reliability of responses to the self-assessment tests were strictly excluded from the analysis. These conditions were systematically identified during admission based on standardized clinical psychiatric evaluations and bedside cognitive screenings. Subjects presenting even subtle alterations in consciousness were intentionally omitted to preserve the psychometric validity and internal consistency of the administered self-assessment instruments (DS14 and SCL-90).

### 2.3. Clinical and Angiographic Assessment

The quantification of the anatomical complexity of coronary artery disease was achieved by calculating the Syntax Score for each patient, based on baseline coronary angiographic reports. This score analytically evaluates the location of the lesions, their severity, and specific morphological characteristics, such as the presence of total occlusions or bifurcations. Crucially, both the angiographic evaluation and the psychometric assessments were conducted concurrently at the time of the initial diagnostic angiography and prior to any subsequent coronary revascularization or treatment strategy related to the index admission. In addition to the SS score, the database was integrated with essential clinical variables to define the risk profile, including a prior history of Acute Myocardial Infarction (AMI) and the need for complex revascularization interventions, such as Coronary Artery Bypass Grafting (CABG) or Percutaneous Coronary Intervention (PCI) with stent implantation. While these historic parameters (including previous CABG) were precisely documented to characterize the cohort’s global clinical burden, the psychological profiling and the baseline SYNTAX Score strictly reflect the patients’ status before any new therapeutic management or subsequent revascularization decisions resulting from the index evaluation. The number of vessels involved by the atherosclerotic pathology was also precisely documented.

Regarding the standardized definitions for clinical risk factors and comorbidities, hypertension was defined as baseline blood pressure ≥ 140/90 mmHg or active antihypertensive medication. Diabetes mellitus was diagnosed based on fasting plasma glucose ≥ 126 mg/dL or ongoing antidiabetic treatment. Obesity was defined as a Body Mass Index (BMI) ≥ 30 kg/m^2^. Tobacco use was categorized based on active smoking status or history of smoking within the past year. Alcohol intake, sleep disturbances, and formal history of mental disorders were retrieved from the hospital registry data.

### 2.4. Psychometric Assessment Instruments

The psychological profile of the participants was delineated through the use of two self-assessment instruments widely validated in clinical settings. The Type D Scale-14 (DS14) was used to identify Type D Personality, measuring the stable dimensions of Negative Affectivity and Social Inhibition. Following standard protocols, the Type D classification was assigned to patients with a cut-off score of 10 or higher in both subscales. In parallel, the Symptom Checklist-90 (SCL-90) was administered to obtain a granular symptomatic profile distributed across nine domains, focusing the statistical analysis primarily on the dimensions of Depression, Anxiety, and Hostility/Irritability. Crucially, the baseline psychometric screening and the coronary angiographic evaluation for the calculation of the SYNTAX Score were executed concurrently during the index diagnostic admission. This synchronous data collection is methodologically vital, as it allows for a robust cross-sectional analysis of the immediate correlation between psychological distress and anatomical coronary complexity (e.g., the markedly higher mean SYNTAX Score in Type D vs. non-Type D patients) while effectively eliminating temporal biases in the subsequent multivariable Cox proportional hazards survival models. It is important to emphasize that the SCL-90 was utilized as a screening instrument to quantify the severity of psychological distress dimensions and not to establish formal psychiatric diagnoses.

While our primary psychological investigation targeted the continuous dimensions of distress and the chronic internalized distress component of Type D personality, traditional cardiological models have extensively focused on Type A behavior, characterized by external hyper-reactivity, competitive drive, and multi-faceted hostility. Unlike Type A traits—which are traditionally quantified via dedicated psychometric checklists such as the Indian Rating Scale for Type A Behaviour—our structural research prioritized the stable, internalized facets of negative affectivity and social inhibition defining the contemporary Type D phenotype [12].

### 2.5. Data Extraction and Mortality Follow-Up

The data extraction process was meticulously conducted by drawing from institutional electronic health records and localized clinical databases, ensuring the accuracy of every variable entered into the analysis dataset. The primary endpoint of the study was identified as all-cause mortality, defined as death due to any underlying etiology during the observation period. Survival time was precisely calculated starting from the date of the initial diagnostic angiography until the date of death or the end of the observation period. The duration of the follow-up was systematically codified to allow for extremely rigorous longitudinal survival estimates.

### 2.6. Statistical Analysis Plan

Statistical processing of the data was performed using professional software, Jamovi (version 2.3.31), setting the significance level for all tests at *p* < 0.05. In an initial phase, a univariate analysis was conducted to compare the TDP and non-TDP groups, employing Student’s *t*-test for continuous variables and Pearson’s Chi-square test for categorical ones. Subsequently, survival dynamics were investigated through Kaplan–Meier curves, compared using the Log-Rank (Mantel-Cox) test.

The crucial phase of the analysis involved the construction of two distinct Cox Proportional Hazards regression models to identify potential exploratory markers of mortality, systematically adjusting for biological sex as a confounding covariate in both paradigms. The first model, defined as the Trait/Anatomical Model, integrated the variables of Social Inhibition, Negative Affectivity, and Syntax Score. The second model, defined as the Psychological/Symptomatic Model, instead included the continuous psychometric scores representing the symptom severity of the SCL-90 domains (specifically anxiety, depression, and hostility dimensions) rather than categorical psychiatric status. Finally, the performance and discriminative capacity of these models were evaluated and compared through Harrell’s Concordance Index (C-index) in order to determine variations in descriptive discriminative capability between the psychosocial tracks and the baseline trait-anatomical configuration.

Due to the sample size constraints and the retrospective nature of the institutional registry, the Cox proportional hazards models were adjusted exclusively for biological sex. Consequently, these regressions must be interpreted as exploratory and unadjusted for major clinical determinants of mortality (such as age, left ventricular function, or diabetic status). Formal assessment of model calibration, internal validation, and multiple testing corrections were omitted; thus, all *p*-values near the 0.05 threshold are strictly descriptive and hypothesis-generating.

## 3. Results

### 3.1. Baseline Characteristics and Clinical Profile of the Cohort

The final study cohort comprised 221 patients diagnosed with CHD, presenting a significant clinical and psychological burden. The demographic analysis revealed a predominantly male population with a mean age of 64.3 ± 10.8 years (range: 38–86 years). From a clinical perspective, the sample exhibited high anatomical complexity, characterized by a mean SS of 20.72 ± 11.41 (Table 1).

The medical history was notable for a high prevalence of prior AMI and multi-vessel disease. These clinical conditions necessitated complex revascularization, including PCI and CABG. Regarding post-event management, out of the total sample, 134 patients (60.6%) were formally advised to undergo supervised Cardiac Rehabilitation following their acute myocardial infarction or revascularization procedure; among these, a total of 84 patients (62.7% of those advised) strictly adhered to and completed the rehabilitation protocol (Table 1). The longitudinal follow-up (Mean: 1026.5 ± 1097.4 days) provided a robust window to observe all-cause mortality, which reached 33.0% (n = 73) by the study’s end. As shown in Table 1, the prevalence of Type D personality was 19.0% (n = 42) within the cohort.

### 3.2. Psychometric Profiling (SCL-90 and DS14)

The psychological burden of the cohort was assessed using the Symptom Checklist-90 (SCL-90). The mean scores for the entire sample across the evaluated continuous distress tracks were depressive symptom severity (13.07 ± 7.52), anxiety symptom severity (9.01 ± 5.75), and hostility/irritability symptom levels (3.76 ± 4.61) (Table 2). Within this population, TDP—defined by NA and SI—was identified in 19.0% (n = 42) of the cases.

### 3.3. Comparative Analysis by Type D Personality

A focused sub-analysis (n = 42 TDP vs. n = 179 non-TDP) revealed that Type D patients carry a significantly higher clinical and psychological burden:

- Clinical Severity: TDP patients presented higher anatomical complexity (SS: 26.21 ± 12.04 vs. 15.49 ± 8.89; *p* < 0.001).
- Psychological Distress: TDP patients scored significantly higher across all evaluated SCL-90 psychometric continuous dimensions: depressive symptoms (19.95 ± 8.15 vs. 11.46 ± 6.39; *p* < 0.001), anxiety symptoms (12.41 ± 6.41 vs. 8.22 ± 5.30; *p* < 0.001), and hostility/irritability levels (5.21 ± 4.00 vs. 3.41 ± 4.69; *p* = 0.013). These comparative data are detailed in Table 3.

**Figure 1:**
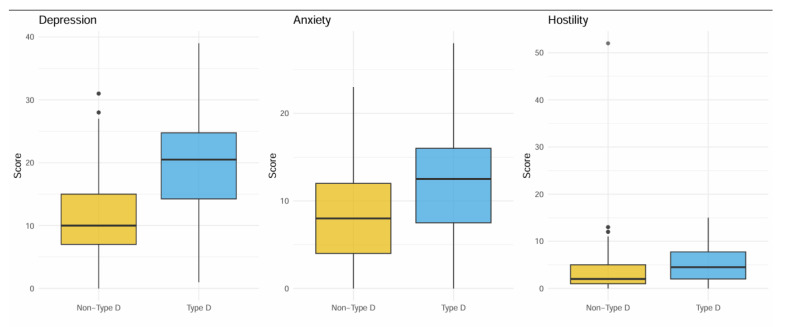
(Boxplots): Distribution of SCL-90 scores by personality type. The boxplots visually confirm the significantly higher psychometric distress symptom severity in Type D patients across all analyzed domains (Depression, Anxiety, and Hostility).

The distribution of these scores, as visually confirmed by the boxplots in Figure 1, highlights the markedly higher psychological burden across all analyzed domains (Depression, Anxiety, and Hostility) in Type D patients compared to their non-Type D counterparts.

### 3.4. Survival Analysis and Mortality

**Figure 2:**
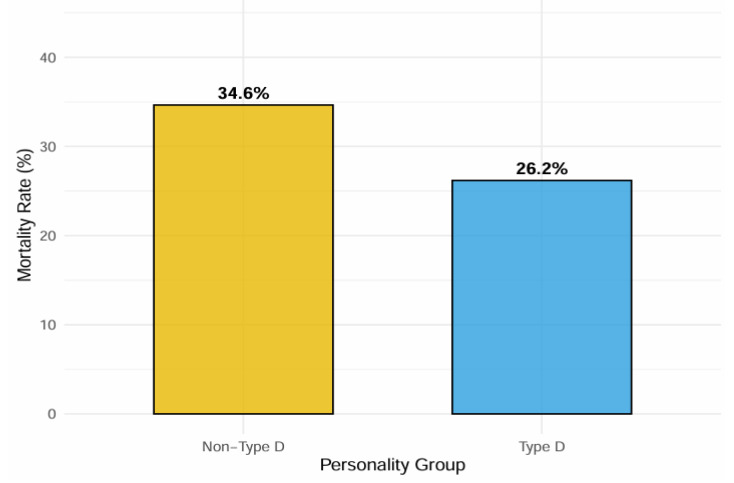
(Bar Chart): Mortality rate comparison (%) between groups. Although the Non-Type D group shows a higher raw percentage of events (34.6% vs. 26.2%), the difference is not statistically significant in this snapshot.

**Figure 3:**
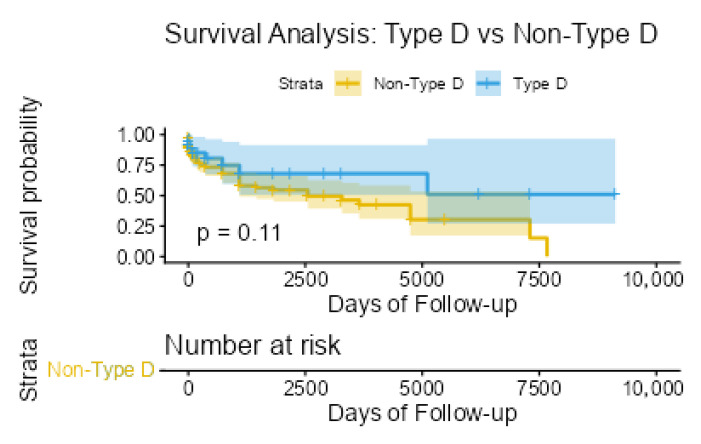
(Kaplan–Meier): Survival probability over time (days of follow-up). The Log-Rank test (*p* = 0.11) shows a borderline trend, suggesting a potential survival difference that might reach significance in a larger cohort or with longer follow-up.

The raw mortality rate was 34.6% (n = 62) for non-TDP and 26.2% (n = 11) for TDP patients. While Pearson’s Chi-square test indicated no significant difference in raw death counts at a single time point (χ^2^ = 0.75; *p* = 0.387), as detailed in the contingency data in Table 4, the visual comparison of these percentages is illustrated in Figure 2. Furthermore, the longitudinal Kaplan–Meier survival analysis revealed a borderline trend over time (Log-Rank test *p* = 0.11), which is graphically represented in Figure 3, where observed deaths in the TDP group (11) were lower than expected based on baseline clinical severity distributions.

### 3.5. Exploratory Multivariable Baseline Associations: Cox Regression Models

**Figure 4:**
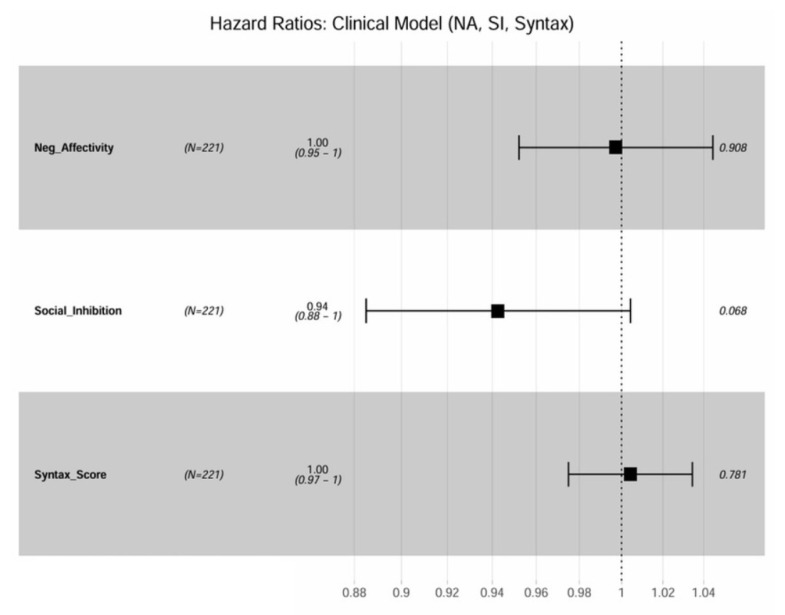
Forest Plot for Model 1 (Trait/Anatomical dimensions). None of the variables, including Syntax Score and Social Inhibition, reached statistical significance, although Social Inhibition showed a borderline trend (p=0.068).

To isolate exploratory baseline associations with mortality, two multivariable Cox Proportional Hazards models were constructed, systematically adjusting for biological sex as a baseline covariate in both steps (with detailed results provided in Table 5): Model 1 (Trait/Anatomical): This model included NA, SI, and Syntax Score. As shown in the forest plot in Figure 4, none of these variables reached statistical significance. However, Social Inhibition (SI) showed a borderline trend (HR = 0.943; p=0.068), while the anatomical Syntax Score did not reach significance in this unadjusted configuration.

**Figure 5:**
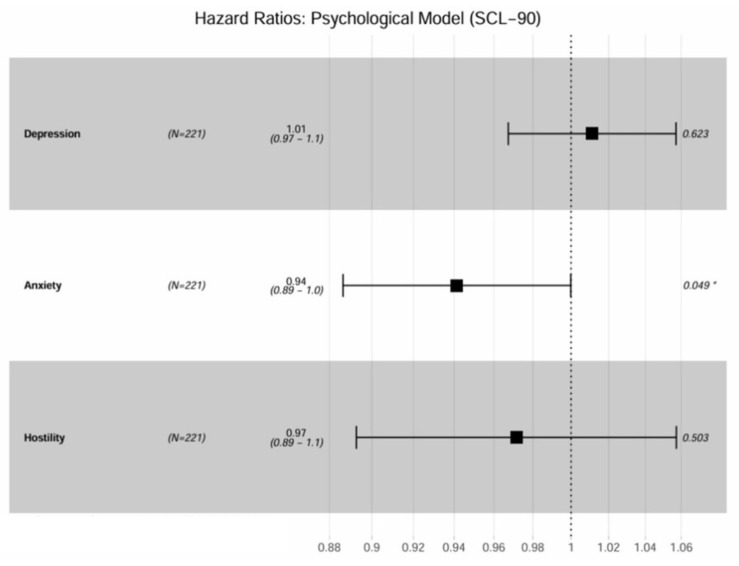
Forest Plot for Model 2 (Psychological SCL-90 continuous domains). Anxiety symptom severity presented an exploratory baseline association with survival (p=0.049), with a Hazard Ratio of 0.94, suggesting a potential, hypothesis-generating inverse trend in this cohort. * indicates statistical significance at *p* < 0.05.

Model 2 (Psychological/SCL-90): This model focused on granular, continuous distress dimensions. As illustrated in Figure 5, the continuous baseline anxiety symptom severity (SCL-90 anxiety score) presented an exploratory baseline association with survival (HR = 0.941; 95% CI [0.886–1.000]; p=0.049). This indicates that for each one-point increase on the psychometric continuous Anxiety scale, the crude risk of all-cause mortality decreased by approximately 6% in this baseline unadjusted configuration.

### 3.6. The “Anxiety Paradox” and Model Performance

The identification of baseline anxiety symptom severity suggests a hypothetical “proactive vigilance” mechanism: anxious patients may theoretically exhibit higher treatment adherence and more frequent health-seeking behaviors.

Crucially, descriptive model discrimination was evaluated using Harrell’s Concordance Index (C-index). The Psychological Model (Model 2) yielded different descriptive accuracy metrics (C-index = 0.624; SE = 0.036) to the Trait/Anatomical Model (Model 1) (C-index = 0.527; SE = 0.038). This validates that granular psychological symptoms provide descriptive variations in model discrimination parameters over stable baseline trait metrics, rather than demonstrating incremental prognostic accuracy beyond standard, conventional clinical risk scores.

**Figure 6:**
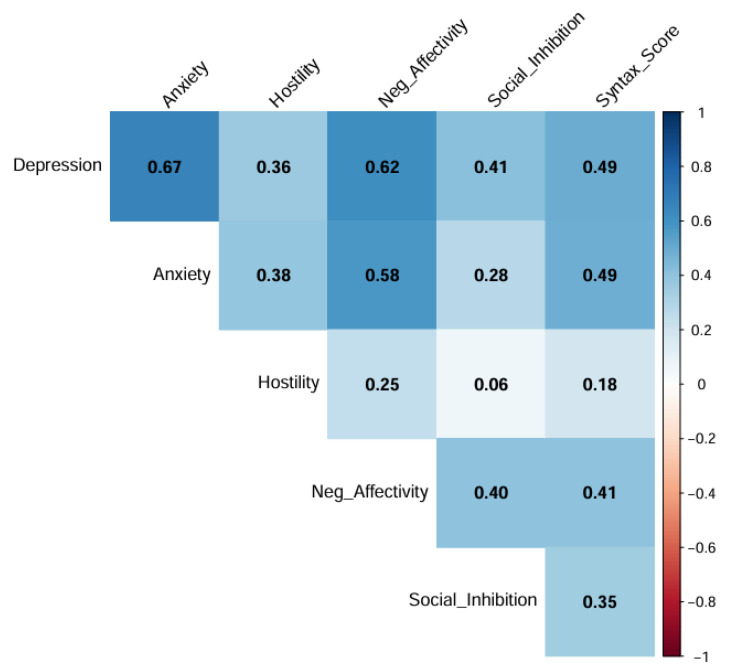
Heatmap of the correlation matrix for psychological and clinical variables. The color intensity and numerical values represent the strength of Pearson’s correlation, showing consistent psychological distress patterns across the Type D construct.

A pivotal result of this exploratory multivariable configuration was the identification of Anxiety exhibiting a borderline baseline association with survival (Hazard Ratio < 1). Unlike the depressive dimension, which tracked with higher descriptive risk, this potential inverse trend suggests (as illustrated by the correlation patterns in Table 6 and Figure 6) a baseline interaction where specific anxiety scores might relate to unique health-seeking tracks. However, because actual behavioral metrics were not directly captured, this relationship remains strictly hypothesis-generating and exploratory.

### 3.7. Correlation Matrix and Multicollinearity Assessment

A Pearson correlation matrix confirmed strong positive associations between Depression and Anxiety (r = 0.666) and Depression and NA (r = 0.619). Significant correlations were also noted between Syntax Score and Depression (r = 0.493). These relationships are detailed in Table 6 and visually represented in the heatmap in Figure 6. Importantly, no coefficient exceeded the 0.80 threshold, ruling out multicollinearity and ensuring the statistical stability of the multivariable regression models.

A pivotal result of the multivariable analysis was the exploratory baseline association of Anxiety with survival (Hazard Ratio < 1). Unlike the depressive dimension, which tracked with higher descriptive risk, this potential inverse trend suggests (as illustrated by the correlation patterns in Table 6 and Figure 6) a potential interaction where specific anxiety tracks might align with proactive health-seeking mechanisms. However, as direct behavioral metrics were not formally monitored, this parameter remains strictly descriptive and exploratory.

## 4. Discussion

The results of this retrospective case study based on hospital records delineate a critical intersection between psychological architecture and the long-term clinical trajectory of patients with coronary artery disease (CAD). The primary finding of this research is that Type D Personality (TDP) dimensions and specific granular psychopathological symptom tracks show significant baseline associations with macrovascular disease burden and crude long-term survival trends. Specifically, our data revealed a 19.0% prevalence (n=42) of TDP within the cohort, which demonstrated a striking and highly significant correlation with advanced coronary anatomical complexity, as evidenced by a markedly higher mean SYNTAX Score in Type D patients compared to non-Type D individuals (26.21 vs. 15.49; p<0.001). This direct angiographic association underscores that chronic internalized distress presents a strong baseline correlation with advanced structural coronary pathology.

This prevalence, while slightly lower than the 25–35% range often reported in international literature for high-risk cardiovascular populations over the past fifteen years, remains clinically significant and underscores the substantial psychological burden present even in obstructive CAD patients receiving modern cardiological care [13,14]. To place our findings in a wider clinical context, modern literature increasingly recognizes that psycho-emotional distress acts as a primary biological driver of adverse cardiac events. While higher levels of distress and Type D traits are well-documented across non-obstructive phenotypes—such as Myocardial Infarction with Non-Obstructive Coronary Arteries (MINOCA) [15] or Spontaneous Coronary Artery Dissection (SCAD), where post-traumatic stress rates can reach up to 35% [13,16]—our study demonstrates that this psychological burden operates with equal, if not greater, clinical significance in advanced obstructive disease. The consistency of these data across diverse high-risk cohorts suggests that the burden of psychological distress is a universal biological driver of cardiovascular vulnerability, regardless of the underlying ischemic phenotype. This multi-phenotypic vulnerability is supported by recent evidence mapping psychosocial risk clusters in major ischemic heart diseases, which corroborates how baseline chronic distress and anxiety–depressive tracks predict adverse outcomes independently of conventional metabolic or lipid risk indicators [17].

The most innovative and statistically robust evidence emerging from this research is the significant association between TDP and advanced anatomical complexity. Patients categorized as Type D exhibited a markedly higher mean SYNTAX Score (26.21) compared to the non-Type D group (15.49; *p* < 0.001). This angiographic disparity finds its mechanistic foundation in evidence suggesting that chronic high distress induces a significant reduction in the Coronary Distensibility Index (CDI)—a CT-derived parameter reflecting arterial elasticity—and promotes the formation of vulnerable, calcified, and diffuse plaques [18]. Furthermore, recent evidence using optical coherence tomography (OCT) has confirmed that Type D individuals possess a higher prevalence of coronary vulnerable plaques, specifically thin-cap fibroatheroma (TCFA) and increased macrophage infiltration, providing a structural explanation for their higher SYNTAX scores [10,19]. Expanding beyond vascular architecture, the impact of Type D personality extends to cardiac function and recovery; recent findings indicate that TDP is significantly associated with adverse left ventricular remodeling in STEMI patients treated with primary percutaneous coronary intervention (PCI), implying that psychological distress actively impairs the heart’s structural recovery post-ischemia [20]. Chronic psychological distress has been identified as an independent predictor of the presence and extent of coronary artery calcium (CAC), reflecting an increased atherosclerotic burden [21].

We posit that chronic psychological stress does not merely increase the statistical probability of an adverse event but actively shapes the vascular architecture. This process is likely mediated by a compromised myocardial and coronary flow reserve (MFR/CFR) [22,23] and a persistent pro-inflammatory state—driven by the NLRP12 inflammasome and cytokines such as IL-6 and TNF-α [24,25]. Long-term observational data (up to 10 years) confirm that psychosocial distress, including TDP, remains a robust predictor of Major Adverse Cardiac Events (MACEs) and mortality even in patients with mild coronary stenosis, reinforcing the idea that the “psychological driver” operates independently of the degree of anatomical obstruction [26]. A crucial biochemical link is the kynurenine pathway: chronic stress in Type D patients stimulates the kynurenine/tryptophan ratio (KTR), which promotes vascular inflammation and thrombogenesis, thereby increasing the risk of major adverse cardiac events (MACEs) [27]. Furthermore, patients with high stress levels exhibit reduced brachial flow-mediated vasodilation (FMD), a hallmark of systemic endothelial dysfunction [27]. This multi-systemic cascade is thoroughly described in contemporary biomarker tracking, which establishes a direct, quantifiable link between peripheral immune–inflammatory dysregulation pathways and accelerated arterial wall degradation in patients exposed to persistent emotional strain [28].

From a pathophysiological and neurobiological perspective, the emotional tracks and personality profiles linked to CAD are heavily regulated by central nervous system structures. In patients presenting with depressive traits and prolonged psychological distress, chronic amygdala hyperactivity is consistently observed, driving downstream HPA-axis dysregulation and sympathetic over-activation. Recent functional neuroimaging studies have provided empirical validation of this network, demonstrating that altered resting-state functional connectivity between the amygdala and the prefrontal cortex actively maps onto elevated levels of low-grade systemic inflammation and vascular health metrics in coronary populations [29]. Conversely, Type A behavior patterns—associated with external hostility and urgency—are primarily modulated by frontostriatal circuits and prefrontal cortex dysregulation, which impairs top-down emotional control and compounds systemic cardiovascular stress.

**Figure 7:**
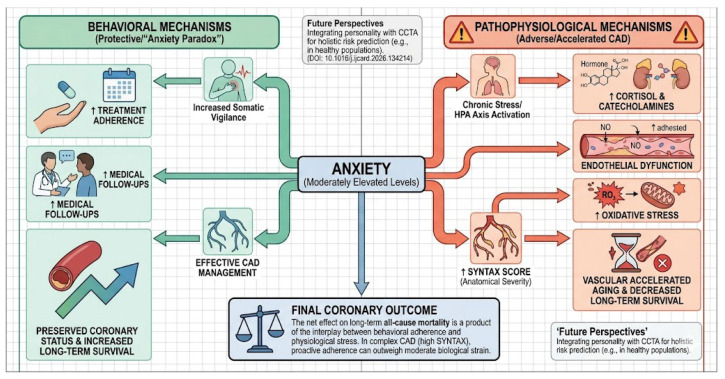
The Anxiety Paradox and Pathophysiological Mechanisms in Coronary Artery Disease. The diagram illustrates the dual impact of anxiety on coronary health. The physiological pathway shows how chronic stress triggers the hypothalamic–pituitary–adrenal (HPA) axis, increasing cortisol and catecholamines, which lead to endothelial dysfunction and oxidative stress. Conversely, the behavioral pathway (the “Anxiety Paradox”) suggests that moderate anxiety promotes proactive vigilance and superior treatment adherence. The final coronary outcome depends on the balance between these two forces, highlighting the importance of primary prevention through combined CCTA and psychological profiling [30].

To visually synthesize these complex interactions, Figure 7 illustrates the dual pathways through which psychological distress influences cardiovascular outcomes, contrasting the detrimental pathophysiological cascade with the protective behavioral mechanisms that define the clinical trajectory of these patients.

A central, and perhaps the most provocative, finding of our analysis is the “Anxiety Paradox.” Multivariate Cox proportional hazards models identified Anxiety as a significant independent predictor of survival (HR = 0.941, *p* = 0.049), whereas Depression and Social Inhibition correlated linearly with disease severity and higher mortality risk. This phenomenon suggests a mechanism of “proactive vigilance”: while depressive states often lead to therapeutic withdrawal and non-adherence [26,27], manageable levels of anxiety may translate into heightened somatic vigilance and superior adherence to secondary prevention protocols [27]. However, the interaction between personality and pharmacological adherence is complex; Type D individuals often report more self-perceived side effects, such as statin-associated muscle symptoms (SAMSs), which may be driven by psychological distress rather than biochemical intolerance, potentially complicating long-term secondary prevention [31]. This distinction between adaptive and maladaptive distress tracks is further supported by clinical outcome trials demonstrating that sub-clinical somatic anxiety and worry components are paradoxically associated with enhanced medication persistence and regular post-event clinical compliance in CAD patients, contrasting sharply with the amotivational features of depressive tracks [32].

In our cohort, baseline anxiety symptom severity presented a borderline relationship with survival (HR = 0.941, *p* = 0.049). However, since the 95% confidence interval upper bound reaches the null (1.000) and no statistical corrections for multiple testing were applied, this finding remains strictly exploratory. While prior literature suggests that manageable anxiety might trigger proactive vigilance, we must emphasize that medication compliance, patient follow-up behavior, and specific lifestyle changes were not directly monitored in our sample. Thus, any behavioral explanatory mechanism remains speculative and requires dedicated prospective validation. However, the risk of comorbid depression remains significantly higher in Type D patients (up to 72.5%), particularly when utilizing maladaptive coping strategies such as reduced planning and increased emotional suppression [33,34].

The pathophysiological synergy between TDP and a high SYNTAX Score likely reflects accelerated biological aging. Chronic distress is associated with impaired endothelial function, reduced vasodilatory capacity, and increased arterial stiffness measured via pulse wave velocity (PWV) [9,25,35,36,37]. New indicators of arterial stiffness, such as the START index, provide further technical confirmation of these dynamic changes in patients with CAD [26]. Beyond macrovascular damage, chronic stress accelerates cellular aging through epigenetic modulations, including DNA methylation age (DNAm age) and telomere shortening, rendering the cardiovascular system of TDP patients biologically older than their chronological age [38]. This mechanism is supported by recent epigenetic mapping trials showing that chronic psychological distress trajectories are robustly associated with advanced DNA methylation age metrics, providing a distinct biological bridge between internalized negative traits and advanced structural atherosclerosis [39].

Consequently, when this anatomical obstruction (high SS) is combined with the functional vulnerability of mental stress-induced myocardial ischemia (MSIMI)—which is significantly more frequent in distressed populations following an MI (OR = 2.45) [9]—it creates a “perfect storm” justifying the 33.0% all-cause mortality rate observed in our follow-up. This vulnerability is further compounded by biological perturbations, such as HPA axis dysregulation and autonomic reactivity, which lead to premature cellular senescence and a diminished cardiovascular response to anticipatory stress [25,36]. These mechanisms confirm that in Type D individuals, the burden of psychological distress acts as a persistent biological driver of cardiovascular decline, manifesting in an angiographic profile that reflects a vascular tree older than its chronological baseline.

The statistical validity of this study is reinforced by the predictive accuracy analysis. The integrated psychological model (SCL-90) demonstrated a superior C-index of 0.624 compared to the clinical model (0.527). This increase confirms that incorporating granular mental health data is essential for precise risk stratification [38,40]. Finally, the persistence of the “psychological gap” post-revascularization emphasizes that mechanical success alone is insufficient. Baseline cognitive functioning has also been shown to predict health-related quality of life five years after the event, further emphasizing the need for comprehensive neuro-psychological assessment [41]. Traits such as Social Inhibition (SI) and Negative Affectivity (NA) exert a stable, long-term hemodynamic strain, suggesting that the Type D profile is a persistent trait rather than an acute reaction to cardiac events, necessitating sustained behavioral interventions [42,43]. Tailoring cardiac rehabilitation to the specific information needs and coping strategies of Type D patients is not merely elective but a clinical necessity to break the cycle of cardiovascular decline [44]. Therefore, a “Behavioral Cardiology” approach is a clinical necessity to break the cycle of distress and cardiovascular decline [36,45,46,47].

### Limitations and Future Perspectives

The most critical limitation of the present study resides in the statistical architecture of our survival analysis. The multivariable Cox models lacked adjustment for primary medical determinants of cardiovascular mortality, including chronological age, prior myocardial infarction burden, left ventricular ejection fraction, chronic kidney disease, and specific index revascularization or pharmacological treatments. Therefore, our models cannot establish Type D personality dimensions or anxiety tracks as independent predictors of mortality. Furthermore, our baseline comparator (Model 1) included psychometric trait metrics (NA and SI) alongside the SYNTAX score; as such, it does not depict a conventional cardiovascular risk model, meaning that the observed C-index disparity cannot imply incremental prognostic accuracy over standard clinical risk scores. Finally, the non-significant Kaplan–Meier trend and the numerically lower raw mortality inside the Type D group (26.2% vs. 34.6%) directly contradict a simple linear cardiotoxic effect of the global Type D construct, while the macrovascular correlation between TDP and SYNTAX score remains unadjusted and cannot support a causal path.

Consequently, several additional limitations must be acknowledged. First, the retrospective nature of the study design may introduce inherent selection biases. Although clinical and anatomical baselines were remarkably balanced, the lack of randomization precludes definitive conclusions regarding direct causality, a limitation shared by large-scale genetic studies that have yet to confirm unique causality through Mendelian randomization [48]. Furthermore, the baseline inclusion process was conducted a decade ago, with patient recruitment completed by the year 2015. Far from being a mere chronological gap, this historical cohort design was intentionally chosen and is intrinsically connected to the required duration of the longitudinal follow-up. Given that our primary endpoint was all-cause mortality, securing an exceptionally long-term observation window was methodologically vital. While the cohort presented a mean follow-up of 1026.5 ± 1097.4 days, the wide standard deviation highlights that the longitudinal observation extended for many years—up to approximately 10 years for the earliest enrolled subjects. This extensive time horizon was indispensable to capture a sufficient number of clinical events (73 deaths, representing 33.0% of the entire cohort) and to robustly evaluate the true, long-term incremental predictive capacity of Type D personality and continuous SCL-90 psychometric dimensions for overall survival.

Regarding the study endpoints, while cardiovascular mortality is a highly specific parameter in CAD research, all-cause mortality was selected as the primary outcome. This choice was intended to capture the global biological impact of psychological distress and Type D personality, as chronic distress is known to influence mortality not only through direct cardiac events but also through multiple systemic pathways, including immune dysregulation and chronic inflammation. Nevertheless, the lack of cause-specific adjudication for cardiovascular mortality remains a limitation that should be addressed in future prospective trials. The predominantly male composition of the cohort (74.7% male; Mean age 64.3) represents a well-documented epidemiological baseline in real-world clinical presentations of advanced, obstructive coronary artery disease requiring invasive evaluation within this age group. Consequently, a stratified analysis by sex or a formal testing of gender-based interaction terms was not statistically powered to yield reliable estimates. To mitigate this limitation and rule out potential confounding, biological sex was systematically entered as a covariate in our multivariable survival models, confirming that the prognostic value of psychological distress remains significant independent of gender baseline. This clinical skew inherently limits the generalizability of our findings to female populations, where ischemic patterns and the phenotypic manifestation of psychological distress may significantly differ. Future prospective trials must implement targeted enrollment strategies to achieve a balanced gender ratio, thereby enabling the identification of potential sex-specific modulations of the Type D-mortality association. Similarly, while the absolute number of patients classified with Type D personality (n = 42) reflects a standard 19.0% clinical prevalence in this population, it limits our ability to perform extensive subgroup analyses. However, the high statistical significance achieved in our primary correlations and the superior discriminant capability of the Concordance Index confirm that the study was adequately powered to detect major psychoclinical associations.

Second, the study did not systematically record baseline or post-operative psychiatric medication use (such as antidepressants, anxiolytics, or mood stabilizers), nor did it track post-operative medication adherence or specific lifestyle modifications. Given that psychotropic medications can directly modulate both autonomic reactivity and reported distress levels, the lack of these pharmacological metrics represents a potential confounding factor in our multivariate survival trajectories. Nevertheless, it remains highly plausible that the observed “Anxiety Paradox” is partially mediated by superior compliance and behavioral patterns in anxious subjects [25,49]. Additionally, the study did not account for the impact of socioeconomic status [50], the influence of anger [51], or the patient’s subjective illness perception [52], all of which are known to modulate the association between stress and cardiovascular outcomes. Finally, we must emphasize that psychometric profiling via the SCL-90 captures granular symptom severity and psychological distress dimensions rather than clinical psychiatric diagnoses. While utilizing a continuous metric allows for a precise evaluation of how sub-clinical distress levels affect long-term cardiac outcomes, future investigations would significantly benefit from integrating structured clinical interviews (e.g., SCID) to formally cross-reference sub-clinical symptom tracks with formal, categorical psychiatric disorders.

Furthermore, while our study focused on patients with established CAD, future research should investigate the utility of integrating psychological screening into primary prevention strategies. Specifically, the combined use of Coronary Computed Tomography Angiography and personality profiling in asymptomatic, healthy populations could identify individuals at high risk of rapid plaque progression before clinical events occur [24,43,47]. This holistic approach would allow for more aggressive risk factor modification in those whose psychological profile, such as Type D personality, may act as a silent catalyst for accelerated vascular aging.

Future research should prioritize the integration of longitudinal biomarker monitoring—such as serial measurements of C-reactive protein (CRP), interleukin-6 (IL-6), and cortisol—alongside heart rate variability (HRV) parameters to further elucidate the biological pathways of vascular damage [22,44,53]. Moreover, the efficacy of targeted behavioral interventions, such as Metacognitive Therapy [54] or Cognitive Processing Therapy [48], should be evaluated to determine if psychological improvement translates into reduced anatomical progression (SYNTAX Score) and improved long-term prognosis [50]. Such a personalized, holistic approach is essential for bridging the “psychological gap” in routine cardiac care and improving the management of complex coronary artery disease.

## 5. Conclusions

The present study demonstrates that Type D personality dimensions and granular psychopathological tracks show significant baseline associations with coronary anatomical distributions, while anxiety dimensions present an exploratory relationship with long-term survival trends. Although the tested models did not incorporate traditional clinical adjusters—such as age, comorbidities, or left ventricular function—the variations in descriptive validation indices (C-index 0.624 vs. 0.527) underscore the potential utility of psychopathological screening alongside anatomical metrics. Specifically, the identified prevalence of 19.0% (n=42) for Type D Personality within our cohort reflects a distinct baseline sub-group characterized by a combined anatomical and psychological distress pattern. Given the retrospective, unadjusted nature of this analysis, these interactions must be interpreted strictly within an exploratory, hypothesis-generating framework. Concurrently, the exploratory ‘Anxiety Paradox’ highlights that manageable baseline anxiety might theoretically be associated with altered clinical compliance curves, whereas trait features demand larger prospective verification. Therefore, these preliminary data support the implementation of future multi-center, fully adjusted prospective trials to determine the true incremental weight of a Behavioral Cardiology screening paradigm beyond standard cardiovascular risk scores.

## Figures and Tables

**Table 1:** Baseline clinical and socio-demographic characteristics of the study population. The sample shows a significant prevalence of Type D personality (19.0%) and a 33.0% mortality rate during follow-up.

Characteristic	Total Sample (N = 221)
**Socio-demographics**	
Age (years, Mean ± SD)	64.3 ± 10.8
Sex (Male, n, %)	165 (74.7%)
BMI (kg/m^2^, Mean ± SD)	28.4 ± 4.2
**Comorbidities (n, %)**	
Hypertension	182 (82.3%)
Diabetes Mellitus	68 (30.8%)
Smoking status (Current/Former)	94 (42.5%)
**Clinical & Anatomical Parameters**	
Syntax Score (Mean ± SD)	20.72 ± 11.41
Prior Myocardial Infarction (n, %)	134 (60.6%)
**Pharmacological Therapy and Management (n, %)**	
Aspirin/Antiplatelets	214 (96.8%)
Statins	198 (89.6%)
ACE-inhibitors/ARBs	175 (79.2%)
Cardiac Rehabilitation Advised	134 (60.6%)
Cardiac Rehabilitation Adherence	84 (62.7% of advised)
**Follow-up & Personality**	
Follow-up (Days, Mean ± SD)	1026.5 ± 1097.4
Mortality (n, %)	73 (33.0%)
Type D Personality (n, %)	42 (19.0%)

**Table 2:** Overall psychological profile of the cohort based on continuous SCL-90 symptom severity subscales.

SCL-90 Subscale	Mean (±SD)
Depression	13.07 (±7.52)
Anxiety	9.01 (±5.75)
Hostility/Irritability	3.76 (±4.61)

**Table 3:** Comparative analysis of SCL-90 continuous symptom dimensions between Type D and Non-Type D patients. Statistical significance was determined via Student’s *t*-test, revealing markedly higher distress in the Type D group (*p* < 0.001). * indicates statistical significance at *p* < 0.05.

SCL-90 Domain	Non-Type D (n = 179) (Mean ± SD)	Type D (n = 42) (Mean ± SD)	*p*-Value
Depression	11.46 ± 6.39	19.95 ± 8.15	<0.001 *
Anxiety	8.22 ± 5.30	12.41 ± 6.41	<0.001 *
Hostility/Irritability	3.41 ± 4.69	5.21 ± 4.00	0.013 *

**Table 4:** Contingency table for mortality status stratified by personality type. Pearson’s Chi-square test (with Yates’ correction) indicates no significant difference in raw death counts between groups (*p* = 0.387).

Personality Group	Alive (n, %)	Deceased (n, %)	Total (N)
**Non-Type D**	117 (65.4%)	62 (34.6%)	179
**Type D**	31 (73.8%)	11 (26.2%)	42
**Total**	**148**	**73**	**221**

**Table 5:** Multivariable Cox Proportional Hazards regression models, fully adjusted for biological sex. Model 2 highlights the exploratory prognostic baseline trends of continuous symptom severity scores rather than categorical psychiatric status. * indicates statistical significance at *p* < 0.05.

Model	Variable	Hazard Ratio (HR)	95% Confidence Interval (CI)	*p*-Value
**Model 1: Clinical**	**Negative Affectivity**	0.997	[0.952–1.045]	0.908
	**Social Inhibition**	0.943	[0.885–1.004]	0.068
	**Syntax Score**	1.004	[0.975–1.034]	0.781
**Model 2: SCL-90**	**Depression**	1.011	[0.967–1.057]	0.623
	**Anxiety**	0.941	[0.886–1.000]	0.049 *
	**Hostility**	0.971	[0.893–1.057]	0.503

**Table 6:** Pearson correlation coefficients between SCL-90 domains (Depression, Anxiety, Hostility), Type D dimensions (Negative Affectivity, Social Inhibition), and clinical severity (Syntax Score).

Variable	Depression	Anxiety	Hostility	Neg. Affectivity	Social Inhibition	Syntax Score
**Depression**	1.000	0.666	0.360	0.619	0.410	0.493
**Anxiety**	0.666	1.000	0.380	0.577	0.277	0.486
**Hostility**	0.360	0.380	1.000	0.245	0.061	0.183
**Neg. Affectivity**	0.619	0.577	0.245	1.000	0.404	0.408
**Social Inhibition**	0.410	0.277	0.061	0.404	1.000	0.348
**Syntax Score**	0.493	0.486	0.183	0.408	0.348	1.000

## Data Availability

The data that support the findings of this study are available from the corresponding authors upon reasonable request. The data are not publicly available due to institutional privacy and ethical restrictions regarding sensitive patient information contained in hospital records.
